# 77680 Nasal Nitric Oxide Levels as a Diagnostic Tool for Primary Ciliary Dyskinesia in Puerto Rico

**DOI:** 10.1017/cts.2021.667

**Published:** 2021-03-30

**Authors:** Wilfredo De Jesus Rojas, Evangelia Morou-Bermudez, Valerie Wojna, Simon Carlo, Ricardo Mosquera

**Affiliations:** 1 University of Puerto Rico, Medical Sciences Campus, School of Medicine, Department of Pediatrics; 2 University of Puerto Rico, Medical Sciences Campus, School of Dental Medicine; 3 University of Puerto Rico, Medical Sciences Campus Department of Internal Medicine; 4 Ponce Health Science University School of Medicine; 5 Department of Pediatrics, University of Texas Health Science Center, Houston Medical School, Houston, Texas

## Abstract

ABSTRACT IMPACT: The implementation of nasal nitric oxide (nNO) as a diagnostic tool to understand the phenotypic/genotypic profiles of Primary Ciliary Dyskinesia (PCD) in Puerto Rico (PR) will be translated in early disease diagnosis, avoidance of comorbidities, and increase survival in our population. OBJECTIVES/GOALS: This study aims to evaluate the role of nNO levels in PCD diagnosis in the Puerto Rican population. Also, we aim to describe the clinical, genetic, and physiological characteristics of PCD in Puerto Ricans to develop a better understanding of the disease. METHODS/STUDY POPULATION: We plan to conduct a cross-sectional study on participants recruited from patients of the Pediatric Rare Lung and Asthma Institute in PR. We will compare nNO levels among genetically confirmed PCD patients, suspected PCD patients with variant of unknown significance (VUS) mutations, suspected PCD patients without genetic mutations, and age-matched healthy subjects. We plan to analyze clinical data and genetic variants to understand the natural history of the disease. The nNO measurements will be completed following previous published protocols. We will also assess the accuracy of the nNO measurements by repeating the measurements two weeks after the initial measurement. RESULTS/ANTICIPATED RESULTS: We hypothesize that many of the VUS present in our population may represent potential new founder mutations not previously reported in the literature. Our expectation is to identify new atypical PCD phenotypes contemplating the heterogenous genetic Puerto Rican pool. We anticipate that nNO levels will help to screen, identify, and confirm diagnosis of patients with clinical PCD in PR. Our findings will be translated in avoidance of further comorbidities and mortality due to earlier disease PCD diagnosis and will expand our genetic understanding about PCD in PR and other diverse populations with heterogenous genetic admixture. DISCUSSION/SIGNIFICANCE OF FINDINGS: We present a significant and novel research proposal that plan to impact the quality of life of patients living with PCD in PR. The implementation of state-of-the-art diagnostic tools like nNO measurement will positively impact and expand our current capabilities to diagnose rare lung diseases like PCD on the island.

